# Impact of I/D polymorphism of angiotensin-converting enzyme (ACE) gene on myocardial infarction susceptibility among young Moroccan patients

**DOI:** 10.1186/s13104-017-3039-1

**Published:** 2017-12-21

**Authors:** Wiam Hmimech, Hind Hassani Idrissi, Brehima Diakite, Farah Korchi, Dalila Baghdadi, Hind Tahri Joutey Hassani Idrissi, Meriem Haboub, Rachida Habbal, Sellama Nadifi

**Affiliations:** 1 Laboratory of Genetics and Molecular Pathology, Medical School, University Hassan II, 19, Street TarikIbnouZiad, B. P: 9154, Casablanca, Morocco; 2 Department of Cardiology, University Hospital Center IbnRochd, Casablanca, Morocco

**Keywords:** Insertion/deletion (I/D) ACE (angiotensin converting enzyme) gene polymorphism, Myocardial infarction (MI), Genetic susceptibility, Moroccan patients

## Abstract

**Objective:**

Our case–control study aimed to access the potential association of insertion/deletion (I/D) ACE (angiotensin converting enzyme) gene polymorphism with myocardial infarction (MI) risk of occurrence among a sample of Moroccan patients, especially young ones.

**Results:**

Distribution of I/D ACE gene variant among cases vs controls, showed that healthy controls carried out higher frequency of wild type allele I compared to cases (23.5% vs 21.79% respectively), when cases were carrying higher frequency of mutant allele D (78.21% vs 76.5% for controls). Patients were-after this- divided into two groups of < 45 and > 55 years of age, to investigate whether or not younger patients carried out higher frequency of the mutant allele D, than older ones. As expected, < 45 years old patients carried out more DD genotype than older ones (68.9% vs 54.6% respectively), and higher frequency of mutant allele D (81.08% vs 75% respectively). Besides, a tendency to a positive association was found under the recessive genetic transmission model (OR [95% CI] = 1.85 [0.93–3.69], P = 0.08), suggesting that the I/D ACE polymorphism may be associated with MI occurrence among younger patients (< 45 years of age).

**Electronic supplementary material:**

The online version of this article (10.1186/s13104-017-3039-1) contains supplementary material, which is available to authorized users.

## Introduction

Myocardial infarction (MI) has always been considered as the most severe complication of coronary artery disease (CAD) worldwide. As being a complex and multifactorial disease, MI involves the interaction of environmental and genetic components [1]. Till now, a wide range of genetic polymorphisms was analyzed for their association with MI risk, such as eNOS gene (endothelial nitric oxide synthase), PAI-1 (plasminogen activator inhibitor type 1), APOA5 (apo-lipoprotein A5), MTHFR (methylene tetra hydrofolate reductase), FII prothrombin, FV leiden and FV Casablanca [2–4], and also gene modulating response to treatment [5–7].

Among the most studied genes is angiotensin converting enzyme (ACE) gene, mapped on chromosome 17q23 (Additional file 1: Figure S1) [8–10]. The gene sequence is composed of 26 exons and 25 introns. The product is a membrane protein involved in hypertension and CAD development [11]. It has been demonstrated that ACE high level was associated with risk of vascular resistance and high blood pressure events, resulting from hyper synthesis of angiotensin II (vasoconstrictor agent) [12]. Angiotensin II is also known to be an essential regulator of proliferation, migration and hypertrophy of vascular smooth muscle cells, increasing thus its association with CAD and MI risk of occurrence [13]. Many SNPs have been described in ACE gene. Interestingly, the insertion/deletion (I/D) in intron 16 was the most investigated. It’s characterized by the insertion or deletion of a 287 noncoding base pair Alu repeat sequence (dbSNP rs4646994), and has been correlated with circulating ACE, intracellular and heart tissue levels [14]. It was reported that DD genotype carriers had increased ACE plasma levels compared to II genotype carriers [15, 16]. Association of I.D ACE polymorphism and MI predisposition has been extensively investigated, but results were divergent and controversial [11].

The main objective of our case–control study was to access whether I/D polymorphism of ACE gene may be associated with susceptibility to MI in a sample of Moroccan < 45 and > 55 years of age, compared to healthy controls.

## Main text

### Methods

Blood samples were collected from 140 unrelated (MI) patients, recruited from the Department of Cardiology, University Hospital Ibn Rochd, Casablanca, Morocco. The control group consisted of 182 unrelated and apparently healthy subjects, with no symptoms of Coronary Artery Disease. Clinical data concerning risk factors and biological parameters were collected for each patient in our study, and an informed consent was developed for both patients and controls. Venous blood from all participants in this study was collected in EDTA tubes. Genomic DNA was extracted from whole blood leukocytes using the salting-out method as previously described by Miller et al. [17].

We used PCR (Polymerase Chain Reaction) to genotype DNA samples for the I/D ACE polymorphism, as previously described by Lindpaintner et al. [18]. Genotyping was performed by amplification from 50 to 100 ng of genomic DNA. PCR gave rise to three profiles: homozygous wild type II (one fragment of 490 pb), ID heterozygous (two fragments of 490 and 190 bp), and homozygous mutant DD (one fragment of 190 pb). The amplified products were separated on 3% agarose gel electrophoresis stained with ethidium bromide (BET), and visualized with UV rayons.

Statistical analysis was performed using SPSS 21. We used Chi square test (χ2) to evaluate the statistical significance of association between genotypes and classical risk factors. Hardy–Weinberg Equilibrium test (HWE) was performed in both cases and controls groups for the analyzed polymorphism. Odds ratio (OR) were calculated to estimate the association between genotypes and MI risk, with a confidence interval (CI) of 95%. Significance was approved at P value less than 0.05.

### Results

I/D ACE polymorphism distribution was in HWE among cases but not controls (Additional file 1: Table S1). Most of our patients were male (62.86%), aged < 45 years of age (76 patients) or > 55 years (66 patients).

Correlation between I/D ACE genotypes and MI risk factors showed was statistically positive with ‘Obesity’, with a tendency to a significant association with ‘gender’ (P = 0.01 and 0.06 respectively) (Table 1).

**Table 1:** Traditional risk factors vs I/D ACE genotypes distribution among MI cases

	MI cases (N = 140)			P value (< 0.05)
	II n (%)	ID n (%)	DD n (%)	
Age (years)				0.1
< 45	5 (6.8)	18 (24.3)	51 (68.9)	
> 55	3 (4.5)	27 (40.9)	36 (54.6)	
Gender				0.06
♂	6 (6.8)	22 (25)	60 (68.2)	
♀	2 (3.8)	23 (24.3)	27 (51.9)	
Hypertension				0.3
Yes	1 (1.9)	17 (32.7)	34 (65.4)	
No	7 (8)	28 (31.8)	53 (60.2)	
Diabetes				0.5
Yes	4 (8.9)	14 (31.1)	27 (60)	
No	4 (4.2)	31 (32.6)	60 (63.2)	
Smoking				0.9
Yes	4 (6.6)	19 (31.1)	38 (62.3)	
No	4 (5.1)	26 (32.9)	49 (62)	
Obesity				0.01*
Yes	3 (12)	13 (52)	9 (36)	
No	5 (4.4)	32 (27.8)	78 (67.8)	
Dyslipidemia				0.6
Yes	1 (2.9)	12 (34.3)	22 (62.8)	
No	7 (6.7)	33 (31.4)	65 (61.9)	
Familial history of CVD				0.6
Yes	0 (0)	4 (40)	6 (60)	
No	8 (6.2)	41 (31.5)	81 (62.3)	

* Statistically significant

We’ve analyzed the same correlation of risk factors and I/D ACE polymorphism distribution, this time among patients < 45 years of age. No statistically significant association was found (Additional file 1: Table S2).

Table 2 shows the allelic and genotypic distribution of I/D ACE polymorphism among cases compared to controls. There was more homozygous wild type profile II among controls than cases (8.8% vs 5.7% respectively), but more heterozygous ID among cases than controls (32.2% vs 29.1% for controls); no difference was found between the two groups concerning the homozygous mutant DD (62.1% for both groups). The transmission models showed no statistical difference between cases and controls (Dominant: OR [95% CI] = 1.7 [0.71–4.06], P = 0.23; Recessive: OR [95% CI] = 1 [0.64–1.58], P = 0.99; Additive: OR [95% CI] = 1.11 [0.77–1.61], P = 0.58).

**Table 2:** Allelic and genotypic distribution of I/D ACE polymorphism among cases and controls

	N = 140	N = 182	OR [95% CI]	P value
	Cases N (%)	Controls N (%)		
II	8 (5.7)	17 (8.8)	1	
ID	45 (32.2)	52 (29.1)	1.84 [0.73–4.66]	0.2
DD	87 (62.1)	113 (62.1)	1.64 [0.67–3.97]	0.27
II^b^	8 (5.7)	17 (8.8)	1	
ID + DD	132 (94.3)	165 (91.2)	1.7 [0.71–4.06]	0.23
II + ID	53 (37.9)	69 (37.9)	1	
DD^a^	87 (62.1)	113 (62.1)	1 [0.64–1.58]	0.99
I allele^c^	61 (21.79)	86 (23.5)	1	
D allele	219 (78.21)	278 (76.5)	1.11 [0.77–1.61]	0.58

^a^Recessive model

^b^Dominant model

^c^Additive model

Doing the same correlation, this time between < 45 and > 55 years of age patients, we found more DD among < 45 years patients than > 55 (68.9% vs 54.6% respectively). The D allele was more frequent among < 45 than > 55 aged patients (81.08% vs 75% respectively). No statistically significant association was found with genotypes or genetic transmission models, even there was a tendency to a positive correlation with the recessive model (OR [95% CI] = 1.85 [0.93–3.69], P = 0.08) (Table 3).

**Table 3:** Allelic and genotypic distribution of I/D ACE polymorphism among < 45 years of age MI patients

	N = 74	N = 66	OR [95% CI]	P value
	< 45 years old N (%)	> 55 years old N (%)		
II	5 (6.8)	3 (4.5)	1	
ID	18 (24.3)	27 (40.9)	0.4 [0.08–1.89]	0.24
DD	51 (68.9)	36 (54.6)		
II^b^	5 (6.8)	3 (4.5)	1	
ID + DD	69 (93.2)	63 (95.5)	1.52 [0.35–6.63]	0.57
II + ID	23 (31.1)	30 (45.4)	1	
DD^a^	51 (68.9)	36 (54.6)	1.85 [0.93–3.69]	0.08
I allele^c^	28 (18.92)	33 (25)	1	
D allele	120 (81.08)	99 (75)	1.43 [0.81–2.53]	0.22

^a^Recessive model

^b^Dominant model

^c^Additive model

### Discussion

Recent reports strongly support the contribution of genetic variations in the pathogenesis of cardiac disorders including myocardial infarction [19]. Angiotensin II produced by ACE is a vasoconstrictor agent, known by promoting smooth muscle cells proliferation and determining structural cardiac changes that could lead to myocardial necrosis and congestive heart failure [20]. Association of ACE with coronary heart diseases (CHD) risk of occurrence has been largely discussed [21].

In Morocco, this is the first case–control study investigating the potential correlation between I/D ACE gene polymorphism and susceptibility to myocardial infarction (MI). 140 MI patients (76 patients < 45 years of age and 64 that were > 55), were compared to 182 healthy volunteers, showing no symptoms of heart diseases, in order to access whether this polymorphism is implicated in MI occurrence in Morocco.

Our results showed that correlation of clinical risk factors with I/D ACE polymorphism was positive with ‘Obesity’ factor (P = 0.01); no more significant association was found with the other risk factors analyzed (Table 1). Reports studying the predisposition to MI have suggests that contribution of genetics as a risk factor for MI occurrence became stronger among younger individuals, having not yet any of the traditional risk factors, such as age, hypertension, dyslipidemia, diabetes and smoking [22]. Trying to test the validity of this suggestion among our studied sample of patients, we performed a statistical correlation between traditional risk factors cited above, with I/D ACE polymorphism distribution especially among < 45 years of age patients (Additional file 1: Table S2). Our results reported no positive association of distribution of this polymorphism with analyzed risk factors among this age range of patients, which goes in accordance with the previous suggestions.

Distribution of I/D ACE gene variant among cases vs controls, showed that healthy controls carried out high frequency of wild type allele I compared to MI cases (23.5% vs 21.79% respectively), when cases were carrying higher frequency of mutant allele D than controls (78.21% vs 76.5% respectively). On the statistical side, no significant difference was found between the two groups (P values > 0.05) (Table 2). Patients were-after this- divided into two groups of < 45 and > 55 years of age, to access whether younger patients carried out higher frequency of the mutant allele D, than older ones (Table 3). As expected, < 45 years old patients carried out more DD genotype than older patients (68.9% vs 54.6% respectively), and higher frequency of mutant allele D (81.08% vs 75% respectively). Besides, a tendency to a positive association was found under the recessive genetic transmission model (OR [95% CI] = 1.85 [0.93–3.69], P = 0.08), suggesting that the I/D ACE polymorphism may be associated with MI occurrence among young patients (< 45 years of age). Unfortunately, on the statistical side, P values were not significant (> 0.05). This may be explained by several factors, such as the limited size of analyzed sample, especially of younger patients (76 patients), the proof is that, when taking < 45 years old patients alone, a tendency to a positive correlation with I/D ACE polymorphism distribution starts to appear (P = 0.08).

Results about the association of this variant of ACE gene with predisposition to MI, are controversial and divergent. Many studies have reported that the DD genotype was associated with MI increased risk of occurrence [16, 19, 23–28], when others suggested the opposite [22, 29–33]. Study conducted by Gardemann et al. [22], reported that DD genotype of ACE gene was associated with Coronary atherosclerosis only among subjects with no classic risk factors, which matches our findings about the tendency of this genotype to be associated with increased risk of MI among individuals without previous classic risk factors (< 45 years of age group of patients).

By another side, there are reasons for cautioning against a direct effect of ACE on heart diseases risk: ACE polymorphisms are associated with ACE plasma activity, but not angiotensin II plasma levels [34, 35]. This may be explained by the fact that rennin is the rate limiting step in angiotensin II production, not ACE [36]. It has been suggested that angiotensin II can affect vascular smooth muscle growth in cell culture [37], however, only hyperplasia is observed in the presence of serum, otherwise, hypertrophy alone does not occur [38, 39].

Previous studies have described a positive correlation between ACE polymorphism and CHD [39–41]. However, these studies have been criticized by a series of opposite studies [32, 33, 42–44]. A large case–control study by Lindpainter et al. [42], has demonstrated no association of D allele with increased risk of ischemic heart disease or MI. The heterogeneity of these results may be explained by numerous factors: differences in the studied populations, or the incapacity to accurately separate cases from controls. In addition, differences between the patient series in the general risk status (diabetes, smoking, and hypertension) may influence the effect of ACE gene polymorphism as a predictor of CHD occurrence. Interestingly, a large meta-analysis has showed that the OR for MI to be associated with the ACE DD is 1.26 [45]. It’s clear that such OR would require larger sample sizes to prove a significant association between ACE polymorphism and risk of MI. in addition, as the frequency of the D allele in the Moroccan population appears to be relatively high (76.5%), a lower predictive effect of the I/D ACE polymorphism in our population would not be surprising. This raises the question of whether this polymorphism is a useful marker to predict the individual risk of MI in a clinical setting.

## Limitations

Our case control study is the first to access whether I/D ACE gene polymorphism may be associated with MI susceptibility in Moroccan population. Our results showed that this variant of ACE gene was not associated with MI risk among the total of analyzed patients (all ages included). However, when differentiating between < 45 and > 55 years of age patients, a tendency to a significant association between the mutant allele D and MI risk started to appear among the younger group of patients (< 45 years of age), clinical risk factors free. One of the limitations of our study is the low sampling. More studies including larger sample sizes and focusing on young patients especially may provide useful help to better understand the contribution of ACE polymorphisms in MI susceptibility.

## Additional file

**Additional file 1: Table S1.** Hardy–Weinberg equilibrium (HWE) among cases and control. **Table** **2.** Traditional risk factors vs I/D ACE genotypes distribution among < 45 years of age MI patients. **Figure S1.** Cytogenetic Location of ACE gene.

## Abbreviations

MI: myocardial infarction
I/D: insertion/deletion
ACE: angiotensin converting enzyme
*: significant
%: percentage
CI: confidence interval
HWE: Hardy–Weinberg Equilibrium
OR: odds ratio
