# Supplementary material for: Impact of I/D polymorphism of angiotensin-converting enzyme (ACE) gene on myocardial infarction susceptibility among young Moroccan patients
Source: BMC Res Notes. 2017 Dec 21;10:763. doi: 10.1186/s13104-017-3039-1 (PMC5740925; doi:10.1186/s13104-017-3039-1)
Supplement: Supplementary file 1 — Additional file 1: Table S1. Hardy–Weinberg equilibrium (HWE) among cases and control. Table 2. Traditional risk factors vs I/D ACE genotypes distribution among < 45 years of age MI patients. Figure S1. Cytogenetic Location of ACE gene. [file 13104_2017_3039_MOESM1_ESM.docx]

**Additional data**

**Additional Table S1: Hardy Weinberg equilibrium (HWE) among cases and controls**

|  | X2 square | P value (P>0.05) |
| --- | --- | --- |
| HWE Cases | 0.45 | **0.79*** |
| HWE Controls | 7.9 | 0.02 |

*** :** Statistically significant

**Additional Table S2: Traditional risk factors Vs I/D ACE genotypes distribution among <45 years of age MI patients**

|  |  | Cases <45 years (N=74) |  | P value (<0.05) |
| --- | --- | --- | --- | --- |
|  | **II n(%)** | **ID n(%)** | **DD n(%)** |  |
| Gender |  |  |  | 0.4 |
| ♂ | 4 (10.3%) | 9 (23.1%) | 26 (66.6%) |  |
| ♀ | 1 (2.9%) | 9 (25.7%) | 25 (71.4%) |  |
| Hypertension |  |  |  | 0.6 |
| yes | 3 (10%) | 7 (23.3%) | 20 (66.7%) |  |
| no | 2 (4.5%) | 11 (25%) | 31 (70.5%) |  |
| Diabetes |  |  |  | 0.09 |
| yes | 4 (15.4%) | 6 (23.1%) | 16 (61.5%) |  |
| no | 1 (2.1%) | 12 (25%) | 35 (72.9%) |  |
| Smoking |  |  |  | 0.9 |
| yes | 2 (7.7%) | 6 (32.1%) | 18 (69.2%) |  |
| no | 3 (6.3%) | 12 (25%) | 33 (68.7%) |  |
| Obesity |  |  |  | 0.8 |
| yes | 1 (7.7%) | 4 (30.8%) | 8 (61.5%) |  |
| no | 4 (6.6%) | 14 (23%) | 43 (70.5%) |  |
| Dyslipidemia |  |  |  | 0.6 |
| yes | 1 (4.5%) | 4 (18.2%) | 17 (77.3%) |  |
| no | 4 (7.7%) | 14 (26.9%) | 34 (65.4%) |  |
| Familial history of CVD |  |  |  | 0.6 |
| yes | 1 (11.1%) | 3 (33.3%) | 5 (55.6%) |  |
| no | 4 (6.2%) | 15 (23.1%) | 46 (70.8%) |  |

*** :** Statistically significant


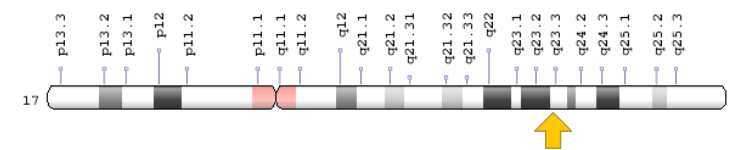


**Additional Figure S1: Cytogenetic Location of ACE gene**

**17:** Chromosome 17; **q23:** long arm of chromosome 17, position 23.
